# Machine learning-mediated *Passiflora caerulea* callogenesis optimization

**DOI:** 10.1371/journal.pone.0292359

**Published:** 2024-01-24

**Authors:** Marziyeh Jafari, Mohammad Hosein Daneshvar

**Affiliations:** 1 Department of Horticultural Science, College of Agriculture, Shiraz University, Shiraz, Iran; 2 Department of Horticultural Sciences, Agricultural Sciences and Natural Resources University of Khuzestan, Mollasani, Iran; Panjab University Faculty of Science, INDIA

## Abstract

Callogenesis is one of the most powerful biotechnological approaches for *in vitro* secondary metabolite production and indirect organogenesis in *Passiflora caerulea*. Comprehensive knowledge of callogenesis and optimized protocol can be obtained by the application of a combination of machine learning (ML) and optimization algorithms. In the present investigation, the callogenesis responses (i.e., callogenesis rate and callus fresh weight) of *P*. *caerulea* were predicted based on different types and concentrations of plant growth regulators (PGRs) (i.e., 2,4-dichlorophenoxyacetic acid (2,4-D), 6-benzylaminopurine (BAP), 1-naphthaleneacetic acid (NAA), and indole-3-Butyric Acid (IBA)) as well as explant types (i.e., leaf, node, and internode) using multilayer perceptron (MLP). Moreover, the developed models were integrated into the genetic algorithm (GA) to optimize the concentration of PGRs and explant types for maximizing callogenesis responses. Furthermore, sensitivity analysis was conducted to assess the importance of each input variable on the callogenesis responses. The results showed that MLP had high predictive accuracy (R^2^ > 0.81) in both training and testing sets for modeling all studied parameters. Based on the results of the optimization process, the highest callogenesis rate (100%) would be obtained from the leaf explant cultured in the medium supplemented with 0.52 mg/L IBA plus 0.43 mg/L NAA plus 1.4 mg/L 2,4-D plus 0.2 mg/L BAP. The results of the sensitivity analysis showed the explant-dependent impact of the exogenous application of PGRs on callogenesis. Generally, the results showed that a combination of MLP and GA can display a forward-thinking aid to optimize and predict *in vitro* culture systems and consequentially cope with several challenges faced currently in *Passiflora* tissue culture.

## Introduction

*Passiflora caerulea* L. is a fast-growing and evergreen climbing plant that can grow as either a shrub or a large tree [1, 2]. The unique secondary metabolite profiles of *P*. *caerulea* such as β-carotene, catechins, tannins, and flavonoids as well as vitamins C and E [3, 4] have resulted in wide use of this plant in traditional medicine due to its anti-addictive [5, 6], anti-hypertensive [7], anti-diabetic/hypolipidemic [8], anti-asthma/anti-respiratory disorders [9], anti-spasmolytic [10], sedative/anti-sleep disorders [11], and anti-depressive/anti-anxiolytic [12, 13] potentials. Since the use of *P*. *caerulea* in medicine has increased over the last several years [4], there is a dire need to develop powerful and reliable biotechnological tools to improve secondary metabolite production in this valuable plant.

**Fig 1:**
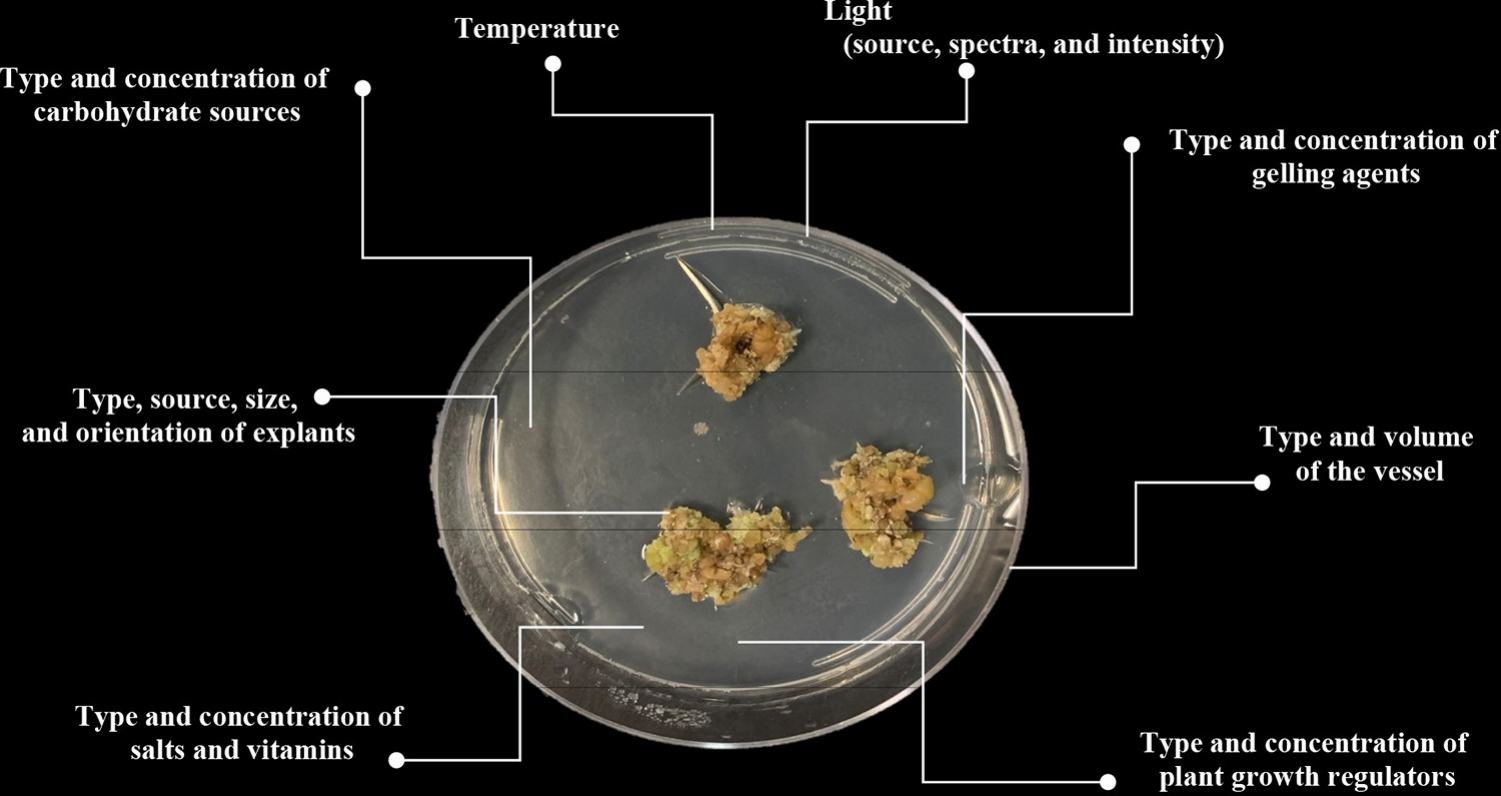
Schematic view of factors influencing callogenesis.

Callogenesis (i.e., *in vitro* callus development) can be considered one of the most powerful biotechnological tools for *in vitro* secondary metabolite production [14–16]. In addition, callogenesis can be used to preserve important genotypes [1], thanks to the possibility of obtaining many clones, banking in a gene bank, and also for bioenergy production [17]. However, it is necessary to optimize several factors involved in callogenesis [18] (Fig 1). The type and concentration of plant growth regulators (PGRs) as well as the type of explants can be considered fundamental factors affecting callogenesis [19]. In fact, any given concentration of PGRs will fall within the various dose-response range according to the species and type of explants [15]. Therefore, the concentration of PGRs should be optimized before their application. However, constructing and optimizing tissue culture protocols represents a major challenge to the field as a whole [20]. Traditionally, tissue culture systems have been developed through large-scale experiments to sequentially optimize individual variables using conventional statistical models and thousands of treatments [21]. These methods are constrained by large treatment requirements and simple linear/curvilinear relationships unsuited for assessing unpredictable interactions of biological systems [21]. Ultimately, such approaches can take insurmountable timespans and resources to develop improved, tough suboptimal tissue culture protocols [22]. Thus, due to the potential to exclude dynamic interactional effects of combined factors, optimization methods must be re-imagined using a modern approach to simultaneously optimize multiple factors for development of precision techniques [23]. For these reasons, applying new powerful approaches for analyzing and predicting *in vitro* culture systems is crucial [18].

Machine learning (ML) is defined as an evolving sub-branch of artificial intelligence which can be considered a reliable and promising computational method to predict and optimize a broad range of complicated biological systems [22, 24–32]. Machine learning offers a new paradigm in the optimization of *in vitro* biology that leverages modern computing power to recognize patterns in complex and chaotic data sets such as those characteristics of tissue culture [18]. The powerful interoperative processes of newly developed nonlinear machine learning algorithms have recently been a focus for plant system biology [28], plant breeding [24], and plant tissue culture [18]. These methods remove uncertainties associated with dynamic tissue responses by diagnosing complex patterns and uses algorithms to predict optimal combinations of factors for desired results [22]. These patterns can then be analyzed using optimization algorithms to predict optimal combinations of factors for desired outcomes [21]. The robustness and accuracy of hybrid ML-optimization algorithms in modeling and predicting different *in vitro* culture systems have been previously confirmed in different species such as chrysanthemum [33–38], passion fruit [39], *Prunus* rootstock [40–42], hazel [43], tomato [44], chickpea [45, 46], wheat [47], cannabis [21, 23, 48–51], and ajowan [52].

In recent years, there has been a growing interest in the integration of genetic algorithm (GA) with artificial neural networks (ANNs) to optimize complex systems, including plant tissue culture systems [24, 29, 42, 53–56]. GA is a powerful optimization technique inspired by the principles of natural selection and evolution, while ANNs are versatile machine learning models that can capture intricate patterns in data [37, 41, 42]. By combining these two approaches, researchers can create a powerful optimization framework to identify optimal combinations of PGRs, nutrient compositions, and other critical factors that influence *in vitro* culture efficiency [18, 57]. The ANN-GA hybrid approach allows for a more systematic and automated exploration of the solution space, leading to improved tissue culture protocols and potentially accelerating the development of desirable plant traits with broader implications for agriculture, horticulture, and biotechnology [18, 53, 57, 58].

Although there is no study to use ANN methods for modeling and optimizing callogenesis of *P*. *caerulea*, ANN can be considered a powerful and helpful approach for getting comprehensive insights into callus formation in this valuable plant. Therefore, in the current study, ML algorithm was employed to develop a predictive model for getting in-depth insight into the effect of PGRs and type of explants on callogenesis of *P*. *caerulea*. Furthermore, GA was linked to the developed ANN model to find the optimized levels of PGRs for maximizing the callogenesis.

## Materials and methods

### Plant material and experimental design

The seed sterilization and germination of *P*. *caerulea* were performed based on our previous protocol [59]. In the current study, three different explants (i.e., leaf, internode, and node) with 0.5 cm lengths were selected from a four-week-old *in vitro*-grown seedling of *P*. *caerulea*. In order to develop callus, leaves were cultured in Murashige and Skoog (MS) [60] medium containing 0.6 g/L agar and 30 g/L (basal media) along with various concentration and types of PGRs on the abaxial side, while internode and node explants were horizontally cultured on the mentioned medium.

The basal media contained various exogenous PGRs at different concentrations including 2,4-dichlorophenoxyacetic acid (2,4-D: 0.0, 1.0, 1.5 and 2.0 mg/L), 6-benzylaminopurine (BAP: 0.0, 0.1, 0.15, and 0.2 mg/L), 1-naphthaleneacetic acid (NAA: 0.0, 1.0, 1.5 and 2.0 mg/L), and indole-3-Butyric Acid (IBA: 0.0, 1.0, 1.5 and 2.0 mg/L). The experiment was performed based on a completely randomized design with a total of 30 treatments in triplicate. Each replicate consisted of 10 culture boxes and one explant was cultured in each box. The pH of all the media was adjusted to 5.7 before autoclaving at 121°C at 0.1 MPa for 20 min. All the chemicals for *in vitro* culture were supplied by Merck (Sigma-Aldrich products, Irvine, UK). The cultures were kept in a growth chamber at the temperature of 25°C ± 2°C in dark conditions for one month. After this period (one month), the callogenesis rate and fresh weight of callus were measured. The obtained data was used as a dataset to feed ML algorithms.

### Machine learning procedures

**Fig 2:**
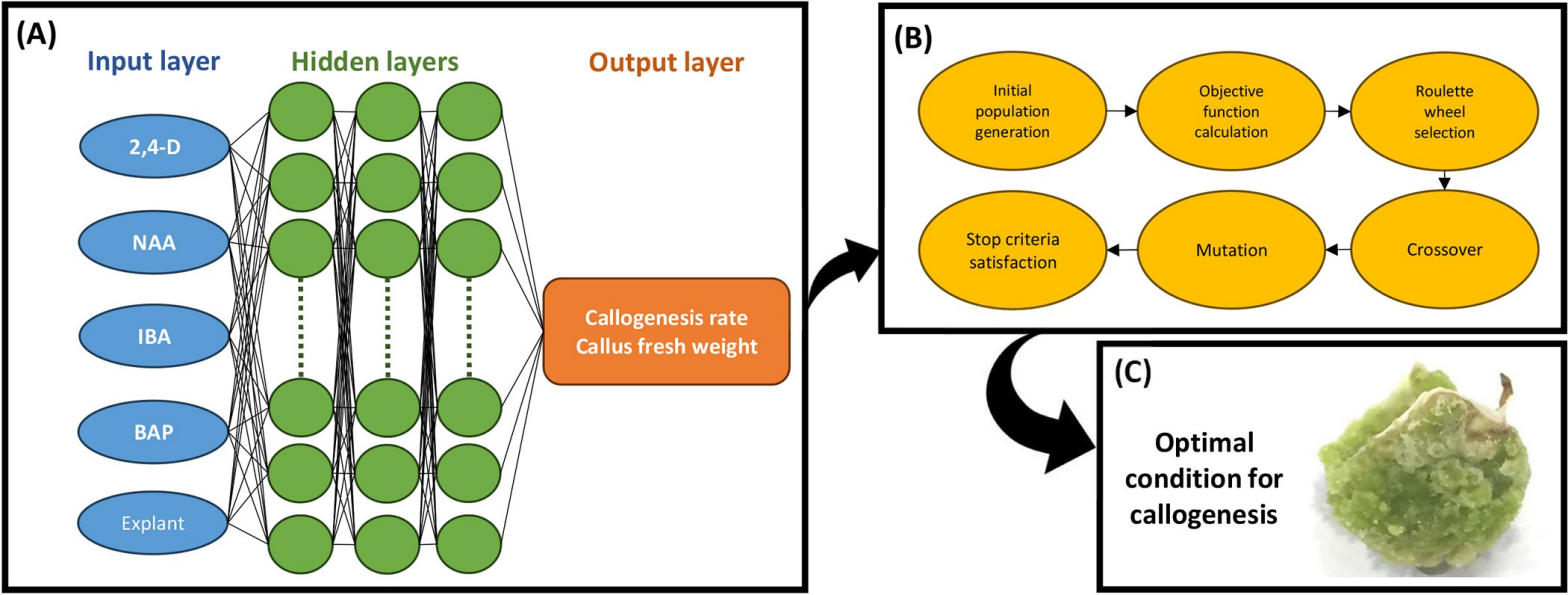
The schematic representation of the step-by-step methodology of the current study including (A) data modeling through multilayer perceptron (MLP) where inputs are explant type, 6-benzylaminopurine (BAP), indole-3-butyric acid (IBA), 2,4-dichlorophenoxyacetic acid (2,4-D), and 1-naphthaleneacetic acid (NAA), and outputs are callogenesis rate and callus fresh weight, (B) optimization process through a genetic algorithm (GA), and (C) optimized callogenesis protocol for *P*. *caerulea*.

Box-Cox transformation was used to normalize data by stabilizing variance and achieving a more approximate normal distribution. It was employed before applying machine learning algorithms to improve model performance. Although principal component analysis (PCA) was applied to determine outliers, no outlier was detected in the dataset. Type of explant (i.e., callus derived from different explants including leaf, node, and internode), 2,4-D, BAP, IBA, and NAA were considered as input variables, while callogenesis rate and fresh weight of callus were fed to ML as target variables (Fig 2). Moreover, 75% and 25% of the dataset were randomly selected to train and test ML algorithms. In the current investigation, a multilayer perceptron (MLP) algorithm was used to model and predict the callogenesis of *P*. *caerulea*.

The MLP-based back-propagation algorithm, one of the most commonly used artificial neural network (ANN) methods, consists of three layers (i.e., input layer, one or more hidden layers, and the output layer). MLP is inspired by the biological neural networks that constitute animal brains (Fig 2A). For the model construction, the MLP algorithm was applied with 3 hidden layers, and the activation function for hidden and output layers was set to hyperbolic tangent sigmoid function (tansig) and linear function (purelin), respectively. The Levenberg-Marquardt algorithm was employed to adjust the bias and weights in the training set of the network. To find the best topology of the model structure, the optimal number of neurons in the hidden layers was detected based on trial-and-error analysis. Additionally, the error was minimized between every input and output variable according to the following equation:

$$Error=\frac{1}{n}\sum_{n=1}^{n}{\left(O_{i}-P_{i}\right)}^{2}$$ (1)

In which, *O*_*i*_ and *P*_*i*_ display the measured values and predicted value, respectively. *n* is the total amount of data.

In an MLP model with *n* inputs and *m* neurons in the hidden layer *P*_*i*_ is obtained from the Eq (2):

$$P_{i}=f\left[\sum_{j=1}^{m}w_{j}.g(\sum_{i=1}^{n}w_{ji}x_{i}+w_{j0})+w_{0}\right]$$ (2)

where *m* is the number of neurons in the hidden layer. *x*_*i*_ and *n* represent the *i*^*th*^ input variable and output variables, respectively. *w*_0_ and *w*_*j*0_ display the bias of output neurons and *j*^*th*^ the neuron of the hidden layer. *f* and *g* denote the transfer functions for the output and hidden layer, respectively. *w*_*ji*_ and *w*_*j*_ indicate the weight connecting the *j*^*th*^ the neuron of the hidden layer and the weight linking the neuron of the output layer.

The accuracy and efficiency of the MLP models were evaluated by using three different performance criteria including coefficient of determination (R^2^), mean absolute error (MAE), and root mean square error (RMSE) according to the following equations:

$$R^{2}=1-\frac{\sum_{i=1}^{n}{\left(y_{i}-{\hat{y}}_{i}\right)}^{2}}{\sum_{i=1}^{n}{\left(y_{i}-{\bar{y}}_{i}\right)}^{2}}$$ (3)

$$MAE=1/n\sum_{i=1}^{n}\left|y_{i}-{\hat{y}}_{i}\right|$$ (4)

$$RMSE=\sqrt{(\sum_{i=1}^{n}{\left(y_{i}-{\hat{y}}_{i}\right)}^{2})/n}$$ (5)

Where *y*_*i*_ is the value of prediction, *n* is the number of data, and ${\hat{y}}_{i}$ is value of observation.

### Optimization process

In the current study, a genetic algorithm (GA), as an evolutionary optimization algorithm inspired by the genetic concepts (Fig 2B), was used to find the optimal level of 2,4-D, BAP, IBA, NAA, and explant type in order to maximize callogenesis rate and fresh weight of callus. Hence, the developed MLP models were fed to GA (Fig 2B) where the generation number, initial population, selection function, cross-over function, crossover rate, mutation function, and mutation rate were respectively considered as 1000, 200, Roulette Wheel, two-point cross-over, 0.6, uniform, and 0.05.

### Sensitivity analysis

Sensitivity analysis was conducted to evaluate the importance degree of explant, 2,4-D, BAP, IBA, and NAA on callogenesis rate and fresh weight of callus by calculating variable sensitivity error (VSE) and variable sensitivity ratio (VSR). VSE shows the RMSE of the developed MLP model when the input is eliminated from the developed model. VSR equals the ratio of VSE and RMSE of the developed MLP when all inputs are available. Then, the importance of input variables is ranked based on the value of VSR. All the analyses were also conducted using MATLAB^®^ software.

## Results

### Effect of plant growth regulators and type of explants on callogenesis in *P*. *caerulea*

**Fig 3:**
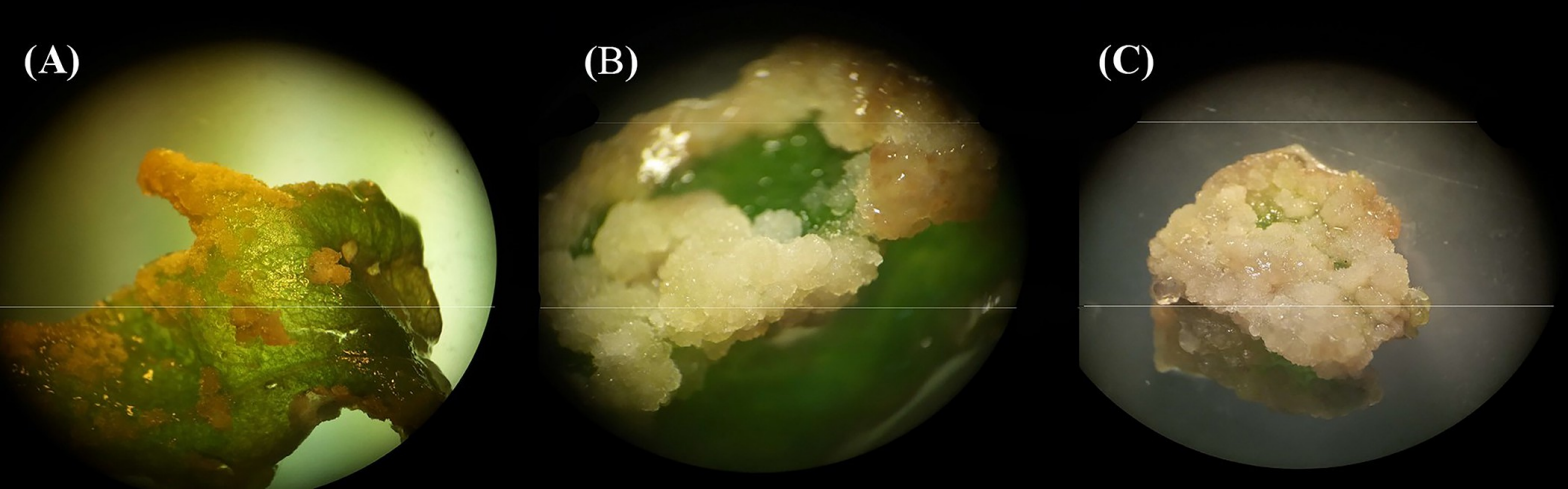
Callus formation in *P*. *caerulea* after (A) one week, (B) two weeks, and (C) one month.

In the current study, the effect of different types and concentrations of PGRs (i.e., 2,4-D, BAP, IBA, and NAA) as well as explant type (i.e., leaf, node, and internode) were evaluated on callogenesis responses (i.e., callogenesis rate and callus fresh weight) of *P*. *caerulea*. Based on our observation, callus formation was initiated after one week (Fig 3A). After two weeks, half of the explant surface was covered by calli (Fig 3B). Ultimately, callogenesis was completed after one month (Fig 3C). Based on Table 1, different callogenesis responses were obtained from different types of explants in the media containing various combinations of PGRs. The highest callogenesis rate and callus fresh weight were obtained from node segment followed by leaf and internode explants (Table 1). In relation to the combination of PGRs, the media containing 2 mg/L 2,4-D along with 0.2 mg/L BAP led to the maximum callogenesis rate and callus fresh weight (Table 1). Also, our results showed that there was no callogenesis in the media without PGRs (Table 1).

**Table 1:** Effect of plant growth regulators and type of explant on callogenesis in *P*. *caerulea*.

Explant type	IBA (mg/L)	NAA (mg/L)	2,4-D (mg/L)	BAP (mg/L)	Callogenesis rate (%)	Callus fresh weight (g)
Leaf	1	0	0	0.1	53.33±3.333	0.53±0.033
Node	1	0	0	0.1	53.33±3.333	0.37±0.033
Internode	1	0	0	0.1	60.00±0.000	0.27±0.033
Leaf	1.5	0	0	0.15	66.67±3.333	0.67±0.033
Node	1.5	0	0	0.15	63.33±3.333	0.50±0.058
Internode	1.5	0	0	0.15	63.33±3.333	0.27±0.033
Leaf	2	0	0	0.2	70.00±0.000	0.73±0.033
Node	2	0	0	0.2	70.00±0.000	0.63±0.033
Internode	2	0	0	0.2	70.00±0.000	0.53±0.033
Leaf	0	1	0	0.1	70.00±0.000	0.77±0.033
Node	0	1	0	0.1	70.00±0.000	0.60±0.058
Internode	0	1	0	0.1	70.00±0.000	0.47±0.033
Leaf	0	1.5	0	0.15	80.00±0.000	0.87±0.033
Node	0	1.5	0	0.15	80.00±0.000	0.63±0.033
Internode	0	1.5	0	0.15	80.00±0.000	0.57±0.033
Leaf	0	2	0	0.2	90.00±0.000	1.73±0.088
Node	0	2	0	0.2	90.00±0.000	1.20±0.115
Internode	0	2	0	0.2	90.00±0.000	1.00±0.058
Leaf	0	0	1	0.1	80.00±0.000	0.73±0.033
Node	0	0	1	0.1	80.00±0.000	0.63±0.033
Internode	0	0	1	0.1	80.00±0.000	0.53±0.033
Leaf	0	0	1.5	0.15	80.00±0.000	0.93±0.033
Node	0	0	1.5	0.15	80.00±0.000	0.77±0.033
Internode	0	0	1.5	0.15	80.00±0.000	0.63±0.033
Leaf	0	0	2	0.2	100.00±0.000	1.87±0.033
Node	0	0	2	0.2	100.00±0.000	1.40±0.058
Internode	0	0	2	0.2	100.00±0.000	1.17±0.033
Leaf	0	0	0	0	0.00±0.000	0.00±0.000
Node	0	0	0	0	0.00±0.000	0.00±0.000
Internode	0	0	0	0	0.00±0.000	0.00±0.000

2,4-D: 2,4-dichlorophenoxyacetic acid; BAP: 6-benzylaminopurine; IBA: indole-3-butyric acid; NAA: 1-naphthaleneacetic acid. Values represent mean ± standard error.

In relation to the interaction between explant type and PGRs, the maximum callogenesis rate (100 ± 0.0%) was observed in all explants cultured in the media containing 2 mg/L 2,4-D along with 0.2 mg/L BAP (Table 1). However, the highest callus fresh weight (1.87±0.033 g) was observed in leaf explants cultured in the media containing 2 mg/L 2,4-D along with 0.2 mg/L BAP (Table 1).

### Evaluation of multilayer perceptron (MLP) in modeling and predicting the callogenesis in *P*. *caerulea*

**Fig 4:**
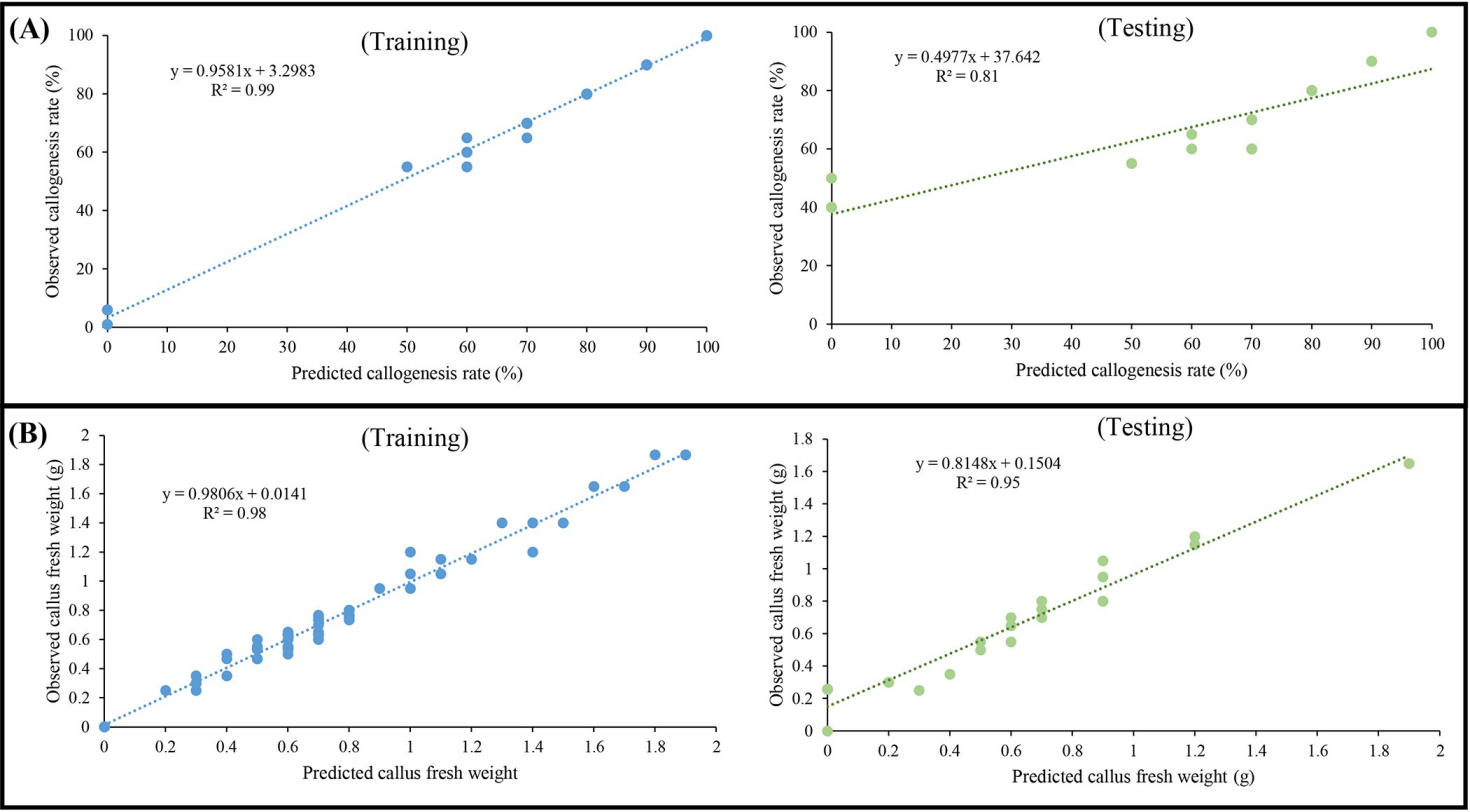
Scatter plot of values of observations vs. predictions in training sets and testing sets of the developed multilayer perceptron (MLP) models for (A) callogenesis rate and (B) callus fresh weight in *P*. *caerulea*.

In the present investigation, the callogenesis responses of *P*. *caerulea* were predicted based on different types and concentrations of PGRs (i.e., 2,4-D, BAP, IBA, and NAA) as well as explant types (i.e., leaf, node, and internode) using MLP algorithm. Based on the results (Table 2), the MLP algorithm led to the development of predictive models with very high R^2^ in either testing or training subsets for all the callogenesis responses including callogenesis rate (R^2^ > 0.81) and callus fresh weight (R^2^ > 0.95). Furthermore, the observed and predicted values in all the callogenesis responses were perfectly correlated in both training and testing subsets (Fig 4).

**Table 2:** Performance criteria of multilayer perceptron (MLP) for callogenesis of *P*. *caerulea* in training and testing subsets.

Output	subset	R^2^	RMSE	MAE
Callogenesis rate	Training	0.996	1.485	0.000
	Testing	0.814	15.591	4.870
Callus fresh weight	Training	0.98	0.06	0.00
	Testing	0.95	0.13	0.04

MAE: mean absolute error; R^2^: coefficient of determination; RMSE: root mean square error.

In addition, RMSE was used to evaluate the accuracy of the developed MLP models. The results showed that the MLP algorithm led to a very high accuracy and performance in either testing or training subsets for all the callogenesis responses including callogenesis rate (RMSE < 15.59) and callus fresh weight (RMSE < 0.13) (Table 2). MAE as another performance criterion showed that the MLP algorithm led to a very high accuracy and performance in either testing or training subsets for all the callogenesis responses including callogenesis rate (MAE < 4.87) and callus fresh weight (MAE < 0.04) (Table 2).

### Optimization process using genetic algorithm (GA)

The developed MLP models were integrated into GA as a single-objective evolutionary optimization method to optimize the concentration of PGRs (i.e., 2,4-D, BAP, IBA, and NAA) and explant types (i.e., leaf, node, and internode) for maximizing the callogenesis responses of *P*. *caerulea*. Based on the results of optimization using MLP-GA (Table 3), the highest callogenesis rate (100%) would be obtained from leaf explants cultured in the medium supplemented with 0.52 mg/L IBA plus 0.43 mg/L NAA plus 1.4 mg/L 2,4-D plus 0.2 mg/L BAP. Also, the maximum callus fresh weight (1.87 g) would be obtained from leaf explants cultured in the medium supplemented with 0.185 mg/L IBA plus 0.15 mg/L NAA plus 1.8 mg/L 2,4-D plus 0.17 mg/L BAP (Table 3).

**Table 3:** Determination of the optimal level of plant growth regulators and explant types for maximizing callogenesis responses through genetic algorithm.

Fitness function	IBA (mg/L)	NAA (mg/L)	2.4-D (mg/L)	BAP (mg/L)	Explant	Predicted-optimized outcome
Callogenesis rate (%)	0.523	0.430	1.400	0.200	Leaf	100
Callus fresh weight (g)	0.185	0.150	1.800	0.170	Leaf	1.87

2,4-D: 2,4-dichlorophenoxyacetic acid; BAP: 6-benzylaminopurine; IBA: indole-3-butyric acid; NAA: 1-naphthaleneacetic acid.

### Importance degree of each input on *P*. *caerulea* callogenesis responses

In the current study, sensitivity analysis through the calculation of variable sensitivity ratio (VSR) was conducted to assess the importance of each input variable (i.e., explant type, 2,4-D, BAP, IBA, and NAA) on the studied objective functions (i.e., callogenesis rate and callus fresh weight). According to our results (Table 4), the explant type was the most important factor for both callogenesis rate and callus fresh weight followed by 2,4-D, BAP, NAA, and IBA respectively. Since the VSR values for explant type are considerably higher than all PGRs, it can be concluded that the explant type is the most important factor for callogenesis (Table 4), showing the explant-dependent impact of exogenous application of PGRs on callogenesis of *P*. *caerulea*.

**Table 4:** Importance degree of each input on *P*. *caerulea* callogenesis responses through sensitivity analysis.

Outcome	Item	Subset	IBA	NAA	2.4-D	BAP	Explant
Callogenesis rate	VSR	Training	1.153163	1.303287	1.47196	1.377404	1.630848
		Testing	0.144687	0.176535	0.190736	0.189839	0.204274
	Rank		5	4	2	3	1
Callus fresh weight	VSR	Training	1.129271	1.423501	1.589794	1.464092	3.263684
		Testing	0.595753	0.705888	0.747405	0.744469	1.535857
	Rank		5	4	2	3	1

2,4-D: 2,4-dichlorophenoxyacetic acid; BAP: 6-benzylaminopurine; IBA: indole-3-butyric acid; NAA: 1-naphthaleneacetic acid; VSR: variable sensitivity ratio.

## Discussion

The micropropagation procedure, as vegetative reproduction in *in vitro* cultures, is an excellent way to obtain clones (i.e., plants genetically identical to the parent plants) [61–63], *in vitro* developmental biology study [64–66], and genetic improvement through genetic engineering approaches [67]. One of the most effective *in vitro* culture methods is callogenesis, where callus is used to obtain *de novo* shoots and/or secondary metabolites [68, 69].

The callogenesis protocol can be divided into two basic phases [15]. In phase I, the plant material is selected [70]. This stage is extremely important because improperly selected explants can determine the results of cultivating [71, 72]. The explants should be taken from a young, healthy plant, living in an optimal environment [73]. Therefore, it is necessary to evaluate the type of explants on callogenesis in phase I [19, 71, 74–78]. Our results showed that the highest callogenesis was obtained from the node explant followed by leaf and internode explants. In line with our results, previous studies showed that node and leaf explants developed a high rate of callogenesis in different *Passiflora* species such as *P*. *alata* [14], *P*. *suberosa* [76], *P*. *setacea* [79], *P*. *mollissima* [80], *P*. *edulis* [81], and *P*. *cristalina* [78].

Then, in phase II, the culture is established. The prepared explants should be transferred to a nutrient medium containing all micro- and macro-elements necessary for the *in vitro* plant’s growth, as well as appropriate exogenous phytohormones determining the direction of development and influencing the physiological processes of explants [19, 82]. The location on the nutrient solution is also important. Explants placed too densely in culture containers may lead to a decrease in the quality of the final product [15]. Our results showed that the media containing 2 mg/L 2,4-D along with 0.2 mg/L BAP led to the maximum callogenesis rate and callus fresh weight. In line with our results, Huh *et al*., [83] compared different concentrations and types of PGRs on callus formation of *P*. *edulis* and reported that that the maximum callogenesis was observed in the medium containing 2 mg/L 2,4-D along with 1 mg/L BAP.

In the initial stage of growth, callus formation can be observed. In this phase, several factors (e.g., type of callus, medium composition, PGRs, light, and temperature) are influenced callogenesis [14, 19, 70, 84, 85]. Although optimizing these factors is a prerequisite for success in indirect shoot regeneration, optimization through conventional statistical methods is a laborious and difficult task due to relying heavily on manual processes of optimization of single factors [39]. Therefore, there is a need to develop and employ novel and innovative computational approaches such as ML for analyzing, predicting, and optimizing callogenesis systems [18]. Using ML algorithms to predict and analyze tissue culture systems are promising to optimize *in vitro* culture procedures [18, 22, 86]. The application of different ANNs is an active area of research in tissue culture [18] which has been used in different systems of *in vitro* culture such as callogenesis [20], shoot proliferation [46], androgenesis [44], somatic embryogenesis [36], and direct shoot regeneration [87].

Therefore, in the current study, MLP as one of the most powerful and well-known ML methods was employed to develop a predictive model for getting in-depth insight into the effect of PGRs (i.e., 2,4-D, BAP, IBA, and NAA) and explant types (i.e., leaf, node, and internode) on callogenesis of *P*. *caerulea*. Our results showed that MLP could be accurately model and predict callogenesis responses (i.e., callogenesis rate and callus fresh weight). In line with our results, previous studies have shown that MLP is a powerful ANN for modeling and predicting different *in vitro* culture systems such as *in vitro* seed germination [50], *in vitro* shoot regeneration [46], shoot growth and development [21], *in vitro* sterilization [33], secondary metabolite production [88], and *in vitro* rooting [40].

Based on the result of sensitivity analysis, the type of explant was the most important factor for all the indirect regeneration parameters, followed by PGRs. It is well-documented that the explant type plays a key role in callogenesis [1, 14, 15]. Indeed, the various *in vitro* responses of each type of explant might be due to the differences in epigenetic regulation as well as endogenous sugars and phytohormones [89]. Similar to our results, previous studies demonstrated that explant type can be considered the most important factor in callogenesis [14, 15, 19]. Due to the totipotent potential of the explant cells, the manipulation of the concentration and ratio of PGRs leads to the differentiation of the explant cells that can ultimately result in callogenesis [90]. Our results revealed that 2,4-D was the second most important factor in callogenesis. In line with our results, previous studies showed that 2,4-D led to a higher frequency of callogenesis compared to other PGRs in different *Passiflora* sp. such as *P*. *edulis* [83], *P*. *suberosa* [76], and *P*. *mollissima* [80].

Traditional approaches to optimizing tissue culture conditions are often time-consuming, resource-intensive, and limited by the complexity of the process [18]. However, the hybrid approach combines the strengths of MLP-based modeling and GA-driven optimization to streamline the optimization process significantly [42, 58]. The hybrid MLP-GA approach presented in this study has proven to be a powerful tool in modeling, predicting, and optimizing the callogenesis process. By incorporating MLP and GA, we have successfully tackled the complexity and nonlinearity inherent in callogenesis. The MLP’s ability to capture intricate relationships between input parameters and callogenesis outcomes has significantly improved predictive accuracy compared to conventional models [52]. The GA’s role in optimization has further demonstrated its effectiveness in rapidly identifying optimal tissue culture conditions, reducing the need for extensive trial-and-error experimentation [40]. Previous studies demonstrated that the combination of these two techniques not only leads to more accurate predictions but also provides a deeper understanding of the underlying mechanisms governing callogenesis [44, 49, 52].

The results of the optimization process (MLP-GA) showed that the maximum callogenesis rate would be achieved from the leaf explant cultured in the medium supplemented with 0.52 mg/L IBA plus 0.43 mg/L NAA plus 1.4 mg/L 2,4-D plus 0.2 mg/L BAP. The result highlighted the importance of balances among PGRs, especially between cytokinins and auxins. In general, a low concentration of cyrokinins and a high concentration of auxins results in callus formation [15, 90, 91]. In line with our results, Huh *et al*., [83] that the maximum callus formation in *P*. *edulis* was obtained from leaf explants cultured in the medium containing a high concentration of auxin (2,4-D) with a low concentration of cytokinin.

## Conclusion

Optimization of callogenesis is one of the key prerequisites for the development of *in vitro* secondary metabolite production and indirect organogenesis protocols in *P*. *caerulea*. Comprehensive knowledge of callogenesis and optimized protocol can be obtained by the application of a combination of ML and optimization algorithms. Our results showed that the callogenesis of *P*. *caerulea* could be precisely predicted and optimized by using ML methods (i.e., MLP) in combination with an evolutionary optimization algorithm (i.e., GA). The optimized PGRs and the suitability of the developed model (MLP-GA) in callogenesis should be assessed by future studies in *P*. *caerulea*. Moreover, the adaptation of a combination of MLP and GA can display a forward-thinking aid to optimize and predict *in vitro* culture systems and consequentially cope with several challenges faced currently in *in vitro* secondary metabolite production.
